# Controllability Analysis of Protein Glycosylation in Cho Cells

**DOI:** 10.1371/journal.pone.0087973

**Published:** 2014-02-03

**Authors:** Melissa M. St. Amand, Kevin Tran, Devesh Radhakrishnan, Anne S. Robinson, Babatunde A. Ogunnaike

**Affiliations:** 1 Department of Chemical and Biomolecular Engineering, University of Delaware, Newark, Delaware, United States of America; 2 Department of Chemical and Biomolecular Engineering, Tulane University, New Orleans, Louisiana, United States of America; Technical University of Denmark, Denmark

## Abstract

To function as intended *in vivo*, a majority of biopharmaceuticals require specific glycan distributions. However, achieving a precise glycan distribution during manufacturing can be challenging because glycosylation is a non-template driven cellular process, with the potential for significant uncontrolled variability in glycan distributions. As important as the glycan distribution is to the end-use performance of biopharmaceuticals, to date, no strategy exists for controlling glycosylation on-line. However, before expending the significant amount of effort and expense required to develop and implement on-line control strategies to address the problem of glycosylation heterogeneity, it is imperative to assess first the extent to which the very complex process of glycosylation is controllable, thereby establishing what is theoretically achievable prior to any experimental attempts. In this work, we present a novel methodology for assessing the output controllability of glycosylation, a prototypical example of an extremely high-dimensional and very non-linear system. We first discuss a method for obtaining the process gain matrix for glycosylation that involves performing model simulations and data analysis systematically and judiciously according to a statistical design of experiments (DOE) scheme and then employing Analysis of Variance (ANOVA) to determine the elements of process gain matrix from the resulting simulation data. We then discuss how to use the resulting high-dimensional gain matrix to assess controllability. The utility of this method is demonstrated with a practical example where we assess the controllability of various classes of glycans and of specific glycoforms that are typically found in recombinant biologics produced with Chinese Hamster Ovary (CHO) cells. In addition to providing useful insight into the extent to which on-line glycosylation control is achievable in actual manufacturing processes, the results also have important implications for genetically engineering cell lines design for enhanced glycosylation controllability.

## Introduction

### Background and Motivation

With a US market exceeding $99 billion in 2011 and an expected steady increase in future sales [1], biopharmaceuticals represent the largest growing class of therapeutics. Many biopharmaceuticals, such as the therapeutic monoclonal antibodies Herceptin and Avastin, are produced as recombinant proteins from Chinese hamster ovary (CHO) cells that are cultivated in bioreactors [2]. As with other manufactured products, these therapeutic proteins are effective only when their product quality attributes (bioactivity, potency, purity, etc.) lie within a specific range of values. Of the many factors that affect the quality and bioactivity of these proteins, arguably one of the most important is glycosylation—a post-translational modification in which a carbohydrate chain, termed a glycan, is added to a protein and modified within the endoplasmic reticulum and Golgi apparatus of a cell [3]–[6]. Due to the pharmacokinetic effects of the various sugar monomers, many therapeutic proteins validated for human use must have a precise distribution of glycans (i.e., specific percentages of glycans with specific sugar monomers such as galactose, sialic acid, or fucose) in order to function as intended *in vivo* [7]. However, unlike other cellular processes such as DNA replication and protein production, glycosylation has no master template. As a result, glycan formation and attachment to the protein are subject to variability and both are often non-uniform. Consequently, regulatory agencies, such as the Food and Drug Administration (FDA) and European Medicines Agency (EMA), are encouraging biopharmaceutical manufacturers to control glycosylation on-line during production [8]. To date, on-line glycosylation control has yet to be implemented in the biopharmaceutical industry for a variety of reasons, mostly attributable to the complexity of these bioprocesses, the non-availability of on-line measurements, and the lack of comprehensive control paradigms tailor-made for such processes.

Before developing and implementing on-line control strategies to address the problem of glycosylation heterogeneity, however, it is imperative first to answer a fundamental question: Is the process of glycosylation intrinsically controllable? This may be stated mathematically as follows: let *x* be the vector of the relative percentages of each glycoform or glycan class; to assess controllability, determine if *x* can be directed from any initial state *x(0) = x_0_* to any arbitrarily specified desired final state *x_f_*, in finite time, via admissible manipulations of available process variables and operating conditions. Such an assessment allows one to determine the degree to which the process of glycosylation can be controlled to yield any desired glycan distribution; it provides a theoretical basis for determining the best achievable control. It is crucial to perform this analysis prior to controller design because even a perfectly designed controller cannot drive the process to the desired set-point if that set-point is intrinsically unachievable.

### Determining System Controllability

Consider a general linear lumped parameter system with *n* outputs, *y*, and *m* inputs, *u*, represented in state-space form as:
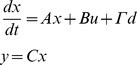
where *x* is the *n_x_*-dimensional state vector, and *d*, the *n_d_*-dimensional disturbance vector. The controllability of such a system is determined directly from the *n* x *nm* “controllability matrix” *L_c_* defined as
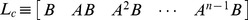

[9]: the process is fully controllable if *L_c_* is of rank *n*; if *L_c_* is not of full rank *n*, then the rank *r (r<n*) of *L_c_* indicates the number of controllable system modes. Such controllability analyses and variations thereof are routinely carried out for chemical processes to assess attainable operation objectives and to improve dynamic performance [10]–[14]. Controllability analysis has also been used for continuous bioreactors to determine control configurations most conducive to reducing the occurrence of bioreactor *washout* (Zhao and Skogestad 1997).

Unfortunately, standard controllability analysis techniques cannot be applied to the glycosylation process. Even though there is sufficient mechanistic knowledge for developing high-fidelity mathematical models of glycosylation (see, for example, the models proposed by Umaña and Bailey [15]; Krambeck and Betenbaugh [16]; Kontoravdi [17]; Hossler et al. [18]; and del Val et al. [19]), the convoluted reaction schemes, complex network architecture of approximately 23,000 reactions, and almost 8,000 resulting glycoforms, combine to dictate that such a mechanistic mathematical model will be exceedingly high-dimensional, complex, and non-linear, and hence severely unwieldy and analytically intractable. It is entirely *impossible* to carry out standard theoretical closed-form, state-space controllability analysis with such models. Assessing the controllability of glycosylation appropriately therefore requires an alternate method.

The approach we propose in this paper is based upon the use of an *appropriately determined* process gain matrix to assess output (rather than state) controllability at operating points of interest. The proposition is predicated upon the fact that for nonlinear processes of practical importance, in a sufficiently small neighborhood of the process operating condition of interest, the map between the input and output variables provides a valid (albeit linearized) representation of the process gain in that region. Also, since in general, the achievable output space is simply a transformation of the permissible input space by the process gain matrix [20], an appropriately determined process gain matrix can be used to assess the controllability of a nonlinear process in any arbitrarily small neighborhood of the operating condition of interest. The primary challenge is therefore two-fold: (i) how to obtain appropriate process gain matrices for exceedingly high-dimensional, extremely complex and highly non-linear systems, and (ii) how to employ the extremely high-dimensional gain matrix to assess controllability.

In what follows, we discuss first a method for obtaining the process gain matrix of highly non-linear systems via model simulations carried out in a systematic and judicious manner according to a statistical design of experiments (DOE) scheme. We then discuss how singular value decomposition of the gain matrix can be used to assess the system's output controllability at the operating point of interest. The practical utility of this method is demonstrated by using it to assess the controllability of various classes of glycans and of specific glycoforms that are typically found in recombinant biologics produced with CHO cells.

## Materials and Methods

### The Controllability Analysis Method

#### Obtaining the Process Gain Matrix

In principle, the *n* x *m* process gain matrix for a process with *n* output variables and *m* input variables can be obtained analytically via a first-order Taylor series approximation of the process model around the steady state of interest. However, such an analytical approach is clearly viable only for a modest-sized system of equations. Because of the sheer complexity of the glycosylation model, especially the extreme high dimensionality, the analytic approach is impractical in this case. Of course, gain matrices can be obtained numerically via simulation, but, for such exceedingly high-dimensional systems, the model simulations must be carried out systematically and judiciously if the required system gain information is to be extracted efficiently from the simulation results.

Thus to obtain the glycosylation process gain matrix, we propose that model simulations be performed according to a systematic statistical design of experiments (DOE) scheme where the combination of input perturbations (factor values) are implemented according to an appropriately chosen experimental design, and the resulting steady state responses analyzed using standard analysis of variance (ANOVA). The rationale is as follows: by definition, the “main effect” of each factor represents the change in the response (process output) resulting from the implemented change in the factor (process input) [21], [22] and is hence directly proportional to what we seek to determine: the gain of the input-output variable pair in question. (In fact, the “factor coefficient”, which for standard factorial experimental designs is exactly half the value of the computed main effect, is precisely the process gain in question.) In this regard, standard ANOVA provides, among other things, estimates of the factor coefficients, 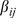 in the equation
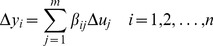
(1)where 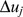 is the change implemented in each input 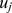 and 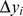 is the change observed in each output 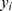. ANOVA also provides the *p*-values associated with each estimated 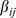, from which statistical significance is determined (for *p*-values less than the defined significance level, typically α = 0.05). The effective *n* x *m* gain matrix can therefore be constructed using the statistically significant 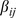
values identified from such analysis of the DOE data, setting all non-significant factor coefficients to zero.

Observe that for nonlinear systems, provided that the magnitude of the perturbations are modest, the resulting factor coefficients provide reasonable estimates of the *local* process gain matrix in the immediate neighborhood around which the input changes were implemented. Such a process gain matrix is therefore expected to change with operating conditions. This is not necessarily a disadvantage of the proposed method; on the contrary, the method in fact allows us to characterize the process gain (and hence output controllability) of the complex nonlinear glycosylation process at various operating regions of potential interest. As we demonstrate later, such results provide insight into the operating conditions around which the process controllability characteristics are most favorable.

#### Assessing Controllability from the Process Gain Matrix

The result from the DOE data analysis described in the previous subsection, which may be represented as:
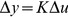
(2)indicates how changes in the process input variables are mapped into changes in the output variables (at steady state) via *K*, the *n* x *m* local process gain matrix, in the operating region around which the simulations were performed. Thus, in principle, this local gain matrix provides direct information about the degree to which each output *y_i_* can be affected by permissible changes in the input variables *u_j_.* In particular, if *K* is square (*m* = *n*) and non-singular, then observe that for any arbitrarily specified desired change 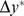 in the vector of output variables, the change in the vector of input variables that will achieve this desired objective is obtained as:
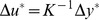
(3)


Thus, under these conditions, the system in question will be fully output controllable if *K* is non-singular (i.e., of full rank). When the gain matrix is non-square (*m*≠*n*), and especially when the dimensionality of *n* and/or *m* is in the hundreds of thousands (as is the case with glycosylation) determining controllability from the gain matrix requires special consideration. First, the extremely high dimensionality presents a practical computational challenge; more importantly, the inequality of *m* and *n*, especially when *n>m*, increases the likelihood that the system will *not* be fully controllable in the region of interest, presenting another practical challenge—that of determining what aspects of the process are controllable (and what aspects are not), and to what extent. As we now show, both practical challenges are handled simultaneously by singular value decomposition of the gain matrix.

The singular value decomposition of an *n x m* matrix *K* is defined as 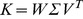
(4)where, for *p* =  min (*m,n*), *Σ* is an *n* x *m* matrix consisting of a diagonal *p* x *p* matrix of the *p* singular values of *K*, *σ_1_*≥*σ_2_*≥… *σ_r_*≥0, *(r≤p*), and *σ_r+1_*  =  *σ_r+2_*  =  …  =  *σ_p_* = 0, augmented with an appropriately dimensioned sub-matrix of zeros; *W* and *V* are orthogonal (unitary) matrices such that *W^T^W  =  WW^T^  =  I*; and *V^T^V  =  VV^T^  =  I,* with *I* as the identity matrix of appropriate dimensions [23]. Here, *r*, the number of non-zero singular values, is the rank of the matrix *K*. If *r = p*, then *K* is of full-rank; it is rank deficient otherwise. Singular value decomposition thus generalizes the invertibility conditions of square matrices to general non-square ones, but for our current purposes of controllability analysis, singular value decomposition does more.

Observe that introducing the singular value decomposition of *K* into Eq. (2) yields
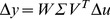
(5)Upon pre-multiplying by *W^T^*, invoking the unitary characteristics of *W*, and introducing new variables defined as
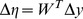
(6)

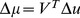
(7)


Eq. (5) is transformed to
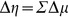
(8)In terms of the original variables, we may now note the following:

(i) Δ*η* is the vector of changes observed in *η =  W^T^y*, a linear transformation of the original output variables, in response to Δ*µ* a change in *µ  = V^T^u*, a different linear transformation of the original input variables. From Eqs. (6) and (7), for each *i* = 1,2, …, *p*

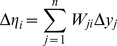
(9)and
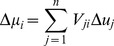
(10)


(ii) Σ is the effective process gain matrix relating the changes in the new output variables *η* in response to changes in the new input variables *µ*. Since Σ consists of a diagonal matrix of the *p* singular values *σ_i_* of the original process gain matrix *K*, the immediate implications are that for each *i*, *i* = 1, 2, …, *p* =  min (*m,n*),
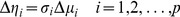
(11)The *p* equations represented by Eq. (11) are central to our proposed method of controllability analysis of glycosylation: they indicate which output modes *η_i_*, a linear combination of the original responses in *y* (in this case the glycoforms or glycan classes), are controllable by which input modes, *µ_i_*, a linear combination of the original inputs in *u* (in this case glycosylation enzymes and sugar nucleotides concentrations), while the value of the associated singular value, *σ_i_* indicates the degree to which (i.e., how strongly) *µ*_i_ affects *η_i_*.

Thus, singular value decomposition of the process gain matrix *K* not only provides the singular values *σ_i_* as a quantitative measure of process controllability, it also indicates which specific output modes are controllable by which specific input modes, in descending order of influence (i.e., the influence of *µ_1_* on *η_1_* is stronger than that of *µ_2_* on *η_2_* which is, in turn, stronger than that of *µ_3_* on *η_3_* …, etc.). While modes associated with zero singular values are entirely uncontrollable, in practice, modes associated with singular values that are smaller than some minimum σ_*_ may be considered as practically uncontrollable since the practical implication of *σ_k_*<σ_*_ is that the associated then *µ_k_* will have minimal effect on *η_k_*,

### Application

To demonstrate the practical utility of the controllability analysis method, it was used to assess the controllability of various classes of glycans and of specific glycoforms that are typically found in recombinant biologics produced with CHO cells. From a process systems engineering perspective, the process of manufacturing appropriately glycosylated proteins is multi-scale in the sense that the sub-processes involved occur on multiple length and time scales, and different sets of variables are of interest at each scale. At the macro-scale, in the bioreactor, one measures and controls bulk bioreactor conditions (pH, agitation, temperature, dissolved oxygen, etc.) and nutrients (glucose, glutamine, etc.); at the meso-scale, within the cytoplasm (or within the cell's membrane boundary), the intracellular nutrients and enzymes are used for primary and secondary metabolism; finally at the micro-scale, primarily within the Golgi apparatus, glycosylation enzymes and sugar nucleotide donors act on proteins during the process of glycosylation. For the purposes of the current discussion, we restrict our attention to the micro-scale, since the variability found in glycan structures actually occurs at this scale. It is important to reiterate why one must determine *first* the extent to which the intracellular process of glycosylation is intrinsically controllable at the micro-scale *before* tackling the practical problem of finding appropriate macro- and meso-scale process variables to control the process of glycosylation. The rationale is simple: if the intra-cellular process is intrinsically *not* controllable, the implication is that no macro- or meso-scale variables will affect changes in the glycosylation process at the micro-scale. In other words, there is no point in seeking process variables to affect the glycan distribution, if there is no way to control the intracellular glycosylation process at the micro-scale. Conversely, establishing controllability implies that effecting intracellular change is possible (at least in principle); then and only then does it make sense for one to seek the practical means for effecting such change via appropriate macro- and meso-scale variables.

The main characteristics of the glycosylation process in question for this specific application example are as follows (See Table 1): the manipulated process variables (factors in DOE terms) are the intra-Golgi concentrations of 15 glycosylation enzymes and sugar nucleotide donors. The cellular outputs (responses) are listed in two categories: (i) the relative percentage of various glycan classes and (ii) the relative percentage of specific glycoforms typically found in biopharmaceuticals. For each cellular output category, we carry out controllability analyses (as explained later in section “Simulation and Data Analysis”) over different operating ranges to determine the effects of process non-linearity on glycosylation process controllability.

**Table 1:** List of responses and inputs used for controllability analysis.

Input (Enzymes & Sugar Nucleotide Donors	Response (Glycan Classes)	Response (Glycoforms)
FucT	S0 †	A1G1S1F
GalT	S1	A2G1S1F
GnTE	S2	A2G2S1F
GnTI	S3	A2G2S2
GnTII	S4	M5
GnTIII	G0	M6
GnTIV	G1	M7
GnTV	G2	M8
ManI	G3	A1
ManII	G4	A1F
SiaT	F0	A2
CMP-SA	F1	A2F
GDP-Fuc		A1G1
UDP-Gal		A1G1F
UDP-Gn		A2G1
		A2G1F
		A2G2
		A2G2F

† Note: The naming convention of the glycan classes is as follows: S# is the number of sialic acid molecules present in the glycoform, G#, galactose, and F#, fucose; where A represents anternarity. For example, the S0 class includes those glycoforms of the 7,565 that are possible with no sialic acid molecules present. The numbers in the glycoform nomenclature represent the number of each sugar molecule attached to the core glycan structure (i.e., three mannose and two n-acetyl glucosamine molecules). For example, the A2G2S2 glycoform has 2 branches each with a galactose and a sialic acid molecule attached to the core glycan structure, as shown in Figure 5.

#### Mathematical Model

The glycosylation model used to illustrate the controllability analysis was based on the Krambeck and Betenbaugh [16] model. The model inputs are 15 glycosylation enzyme and sugar nucleotide donor concentrations in the Golgi apparatus as specified in Table 1; the outputs are the concentrations of 7,565 glycoforms. Only those glycosylation reactions that occur within the compartments of the Golgi apparatus were included in the model. The sequential enzymatic reactions of the glycosylation process were simulated via a reaction network, with each reaction in the network constructed from the set of reaction rules described in Krambeck and Betenbaugh (2005) for identifying the glycosylation enzymes and how these enzymes act upon each glycoform. (For example, one of the reaction rules states that the enzyme ManI cleaves a mannose nucleotide if there are more than five mannose groups on the glycan.) The trans-Golgi network and stacked cisternae (cis, medial, and trans) of the Golgi were modeled as four compartments with each treated like a well-mixed reactor. Consequently, at steady state, the glycoforms satisfy the following mass balance equation: 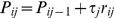
(12)where P*_ij_* is the concentration of glycoform species *i* in compartment *j*, *τ_j_* is the residence time of compartment *j,* and *r_ij_* is the net rate of production of glycoform *i*, defined as:
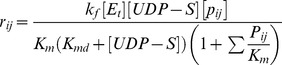
(13)


Here *E_t_* is the glycosylation enzyme concentration, *UDP-S* is the sugar nucleotide donor concentration, *k_f_, K_md,_* and *K_m_* are glycosylation enzyme kinetic parameters. Because we are interested here only in the steady state glycoform concentration profile (i.e., [P_ij_] as *t*→∞), we set *r_i_* = 0 and solved the resulting system of equations for [P_ij_]. The resulting species concentration data were then used to calculate the relative percentage of the glycan classes or specific glycoforms in Table 1.

#### Simulation and Data Analysis

As described in section “Obtaining the Process Gain Matrix”, the glycosylation process gain matrix required for the controllability analysis was obtained from glycosylation model simulations carried out according to a DOE scheme. Since the model inputs are 15 enzyme and sugar nucleotide donor concentrations, we employed a 2^15-9^, resolution IV fractional factorial experimental design, which allows us to avoid confounding enzyme main effects with potentially interesting 2-factor interactions (see, for example, Box et al. [24] or [21].

The implemented fractional factorial design assumes a linear (or at best bilinear) representation of the process within the specified process operating range. Since glycosylation is a nonlinear process, controllability analyses were performed over multiple operating ranges to determine the effect of process non-linearity on controllability. For purposes of illustration, we chose to examine glycosylation controllability over the three operating ranges of cellular conditions shown in Table 2. Range 1 represents the widest possible, physiologically relevant range of intracellular glycosylation enzyme and sugar nucleotide concentrations as determined from the literature. Range 2, a lower subset of the physiologically relevant range, includes concentrations that are within ±0.5 µM of the nominal values used by Krambeck and Betenbaugh [16]. Range 3, an upper subset of the physiologically relevant range, was obtained as 1.75 times the nominal values used by Krambeck and Betenbaugh (2005) ±0.5 µM.

**Table 2:** Operating ranges of input factors used in controllability analysis (i.e., µM concentrations used for each glycosylation enzyme and sugar nucleotide donor investigated as factors in DoE).

	Range 1		Range 2		Range 3	
Factor	Low	High	Low	High	Low	High
**FucT**	0.2	8.5	1.25	3.75	3.75	6.25
**GalT**	0.2	8.5	0.33	0.99	0.99	1.65
**GnTE**	0.2	8.5	1.735	5.20	5.20	8.67
**GnTI**	0.2	8.5	1.52	4.57	4.57	7.62
**GnTII**	0.2	8.5	0.64	1.93	1.93	3.22
**GnTIII**	0.2	8.5	0.55	1.65	1.65	2.75
**GnTIV**	0.2	8.5	1.81	5.43	5.43	9.05
**GnTV**	0.2	8.5	0.20	0.60	0.60	1.00
**ManI**	0.2	8.5	0.89	2.67	2.67	4.45
**ManII**	0.2	8.5	0.66	1.98	1.98	3.30
**SiaT**	0.2	8.5	0.50	1.50	1.50	2.50
**CMP-SA**	960	7200	1200	3600	3600	6000
**GDP-Fuc**	1000	7500	1250	3750	3750	6250
**UDP-Gal**	1520	11400	1900	5700	5700	9500
**UDP-Gn**	3680	27600	4600	13800	13800	23000

Using the model described in section “Mathematical Model”, simulations of MAb glycosylation were carried out according to the 2^15-9^ fractional factorial design over the three operating ranges in Table 2. Estimates of factor coefficients and the associated *p*-values were obtained from the resulting simulated glycan data using standard fractional factorial ANOVA in Minitab16. The process gain matrix, *K,* was constructed from factor coefficient estimates, retaining statistically significant values (for which *p*<0.05) and setting the others (for which *p*>0.05) to zero. Finally, singular value decomposition was performed on the resulting process gain matrix *K* to produce the singular value matrix, Σ, and unitary matrices *W* and *V^T^* from which the orthogonal output modes *η_i,_* and orthogonal input modes, *µ_i_*, were constructed according to Eqns. (9) and (10).

## Results and Discussion

### Controlling Glycan Classes

**Figure 1:**
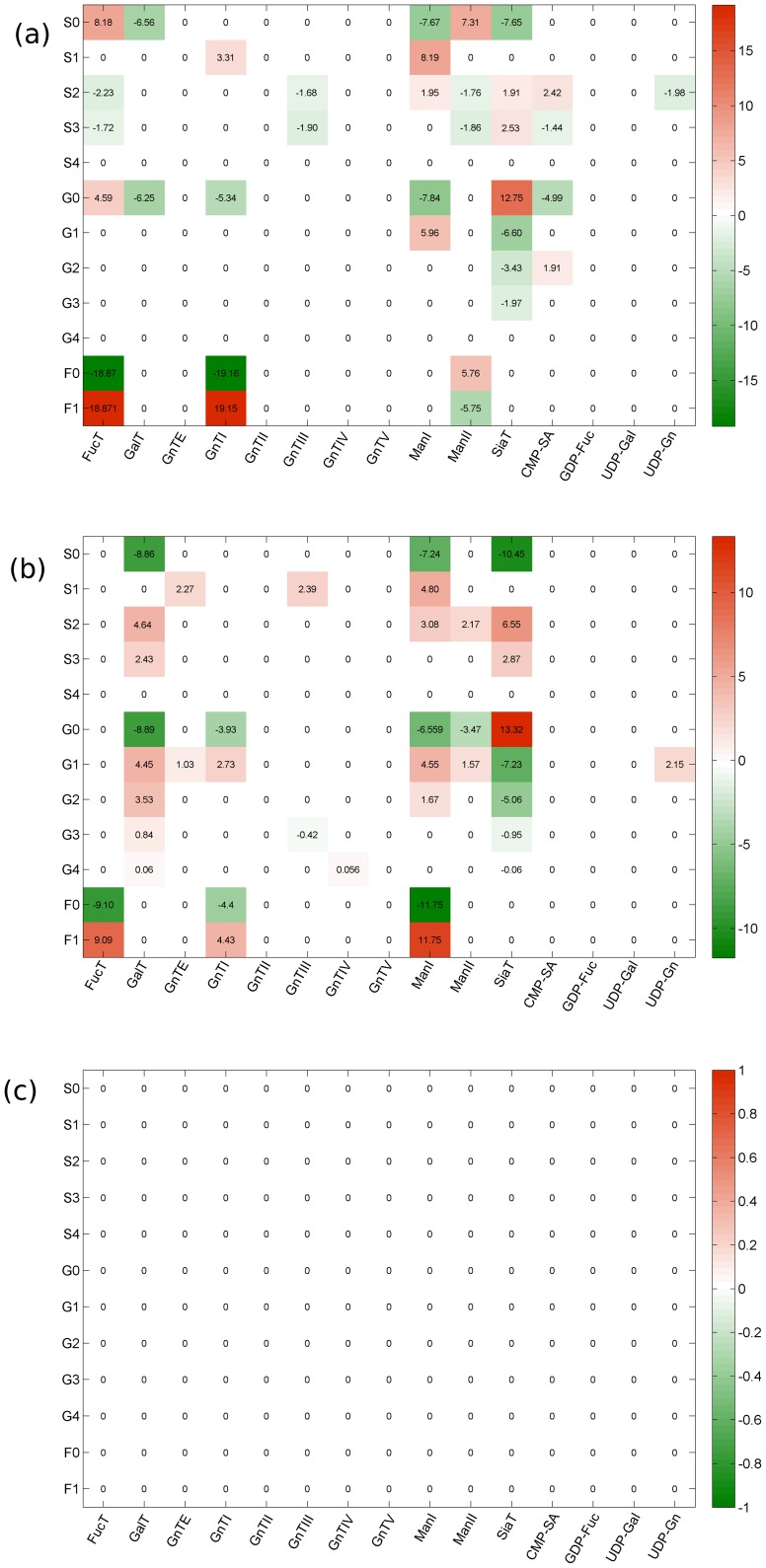
Heat maps representing the significant elements of the process gain matrices for the glycan classes in operating (a) Range 1, (b) Range 2, and (c) Range 3. Visual inspection suggests that significant process gains are associated with 10 of the 12 glycan classes when the process is operated in Range 1 or 2 indicating that the relative percentage of glycan classes can be changed in these operating ranges. There are no significant process gains for any glycan class in operating Range 3, suggesting that the relative percentage of glycan classes cannot be affected or controlled at all when the process is operated in Range 3.

Visual inspection of the process gain matrix can provide some intuition about the potential controllability of glycosylation at the micro-scale. This is because only those process output variables connected via significant, non-zero elements of the gain matrix to process input variables (sugar nucleotides and glycosylation enzymes) can be affected by manipulating the input variables, and thus are potentially controllable. The heat maps of the process gain matrices shown in Figure 1 indicate that significant process gains are associated with 10 of the 12 glycan classes when the glycosylation process is operated within the values in Range 1 or 2 for the intracellular variables. On the other hand, there are no significant process gains associated with any glycan class in operating Range 3, indicating that the relative percentage of glycan classes cannot be changed to any significant extent when the process is operated in Range 3. Such a visual inspection of the process gain matrices can provide no more than a general sense of potential controllability however; determining precisely which specific modes are actually controllable, and the extent to which each is controllable, requires singular value decomposition of the process gain matrix, as described below.

#### Identifying controllable modes

**Figure 2:**
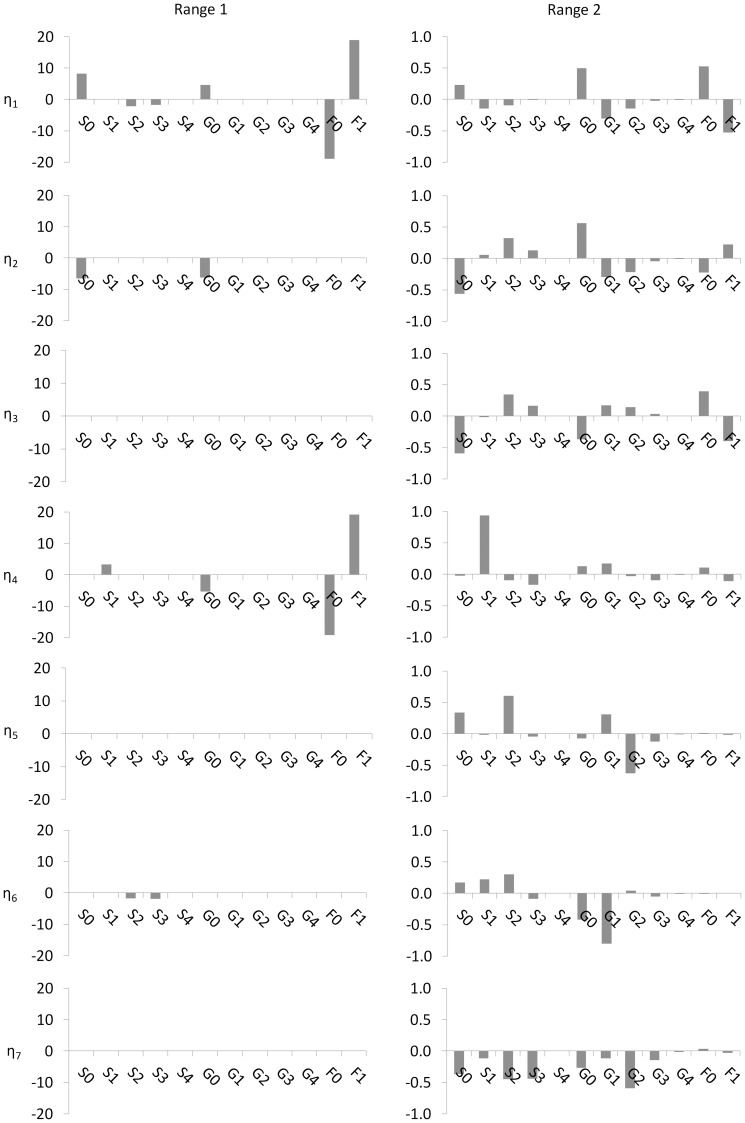
Graphical representation of the coefficients associated with each glycan class in the controllable output modes, *η_i_.* Modes that were not controllable (i.e. associated with singular values, ***σ_i_***<σ_*_ = 1) are not shown. Each column shows the glycan classes (output modes) that are controllable in each operating range (See Table 1). No output modes are shown for operating Range 3 since no controllable modes were found in this range. Coefficients were obtained using eq. 9 following singular value decomposition of the glycan class process gain matrix as described in section “Assessing Controllability from the Process Gain Matrix”. How much the glycan class contributes to an output mode is reflected in the coefficient associated with that variable in the linear combination. A dominant contributor to a mode (where one exists) is identified by the variable with the largest coefficient in the weighted sum. Any glycan classes associated with a non-zero coefficient can be affected by perturbations in the associated input mode; however the dominant glycan class will be affected the most.

**Figure 3:**
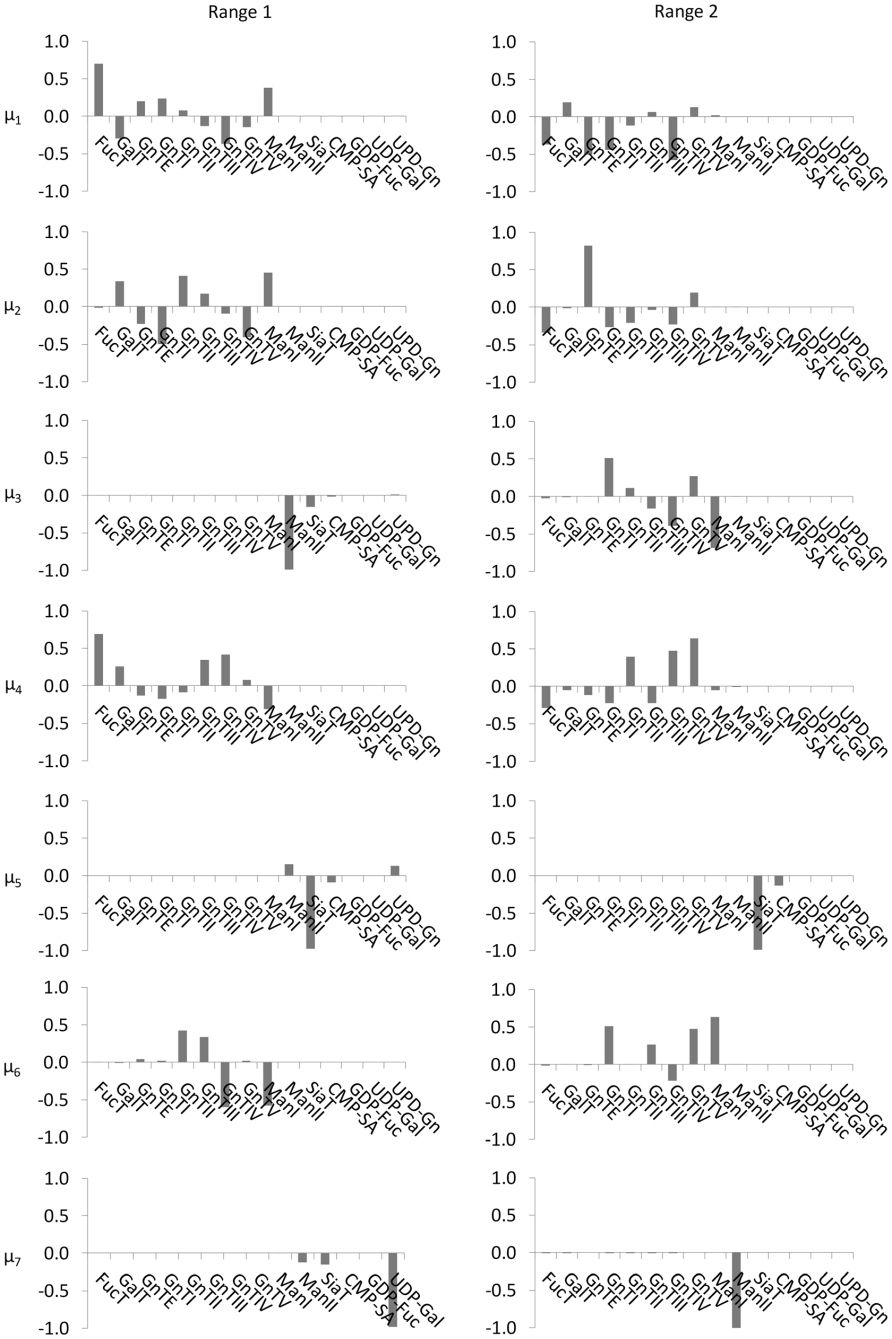
Graphical representation of the coefficients associated with each enzyme and sugar nucleotide donor in input modes, *µ_i_*, associated with the controllable output modes for glycan classes, *η_i_,* shown in Figure 2. Each column shows the coefficients associated with the enzymes and sugar nucleotides of each input mode in each operating range (see Table 1). No input modes are shown for operating Range 3 since no controllable modes were found in this range. Coefficients were obtained using eq. 10 following singular value decomposition the process gain matrix for the glycan classes as described in section “Assessing Controllability from the Process Gain Matrix”. How much the enzyme or sugar nucleotide contributes to an input mode is reflected in the coefficient associated with that variable in the linear combination. A dominant contributor to a mode (where one exists) is identified by the variable with the largest coefficient in the weighted sum. The enzyme(s) and/or sugar nucleotide(s) that are dominant contributors of the input mode affect the glycan classes of the associated output mode the most.

As discussed in section “Assessing Controllability from the Process Gain Matrix”, the magnitude of each singular value, *σ_i_*, provides a quantitative measure of the relative extent to which each output mode, *η_i_*, is controllable by the associated input mode, *µ_i_*. Relatively large values of *σ_i_* indicate that the corresponding mode *η_i_* is relatively more controllable (i.e., perturbations in *µ_i_* will result in more noticeable substantial changes to *η_i_*) than modes associated with smaller values of *σ_i_*. The results of the singular value decomposition of the process gain matrices in Figure 1 are the singular values, *σ_i_*, presented in Table 3, and the coefficients associated with the controllable output and input modes, *η_i_* and *µ_i_*, shown in graphical form in Figure 2 and Figure 3, respectively. Here, output modes *η_i,_* associated with singular values, *σ_i_*≥σ^*^ = 1, are considered controllable. The choice of the threshold value σ^*^ = 1 is somewhat arbitrary, informed in this specific case by the consideration that for those modes associated with *σ_i_*<1, changes to *µ_i_* will *not* produce changes in *η_i_* that are large enough (by comparison) to be of practical consequence in a process control scheme.

**Table 3:** Singular values, *σ_i_*, obtained from singular value decomposition of the glycan class process gain matrices for three operating ranges.

Singular value	Range 1	Range 2	Range 3
**σ_1_**	39.2	25.9	0
**σ_2_**	21.1	20.4	0
**σ_3_**	17.0	14.3	0
**σ_4_**	6.9	4.5	0
**σ_5_**	3.9	2.5	0
**σ_6_**	3.1	2.1	0
**σ_7_**	2.3	1.0	0
**σ_8_**	0.7	0.8	0
**σ_9_**	0.2	0.3	0
**σ_10_**	0	0.1	0
**σ_11_**	0	0	0
**σ_12_**	0	0	0

Output modes associated with singular values, *σ_i_*>σ_*_ = 1, are considered controllable.

When the glycosylation enzyme and sugar nucleotide donor concentrations are restricted to the Range 1 and 2 values, 7 of the 12 modes are associated with singular values *σ_i_*≥1 (See Table 3). This indicates that 7 output modes (linear combinations of glycan classes) are controllable with the intracellular concentrations of glycosylation enzymes and sugar nucleotides specified by the associated input modes. The singular values associated with operating Range 3 indicate that no mode is controllable in this operating range. These results are consistent with the preliminary assessment of controllability based on visual inspection of the gain matrix; however, the singular value decomposition results provide additional quantitative information regarding not just the controllable modes but also the extent of controllability of each mode—information that is not possible from mere inspection of the process gain matrix. That is, while 7 output modes are controllable over the indicated operating ranges, the extent to which each is mode is controllable (indicated by the relative magnitude of its corresponding *σ_i_*) is different in each case. For example, in Range 1 the first output mode, *η_1_*, with associated singular value, *σ_1_* = 39.2, is the most controllable of the glycan class output modes. On the other hand, with a singular value, *σ_7_* = 2.3, the seventh output mode is the least controllable of the glycan class output modes in this operating range. This information indicates that in controlling the seventh output mode, *η_7_*, within operating Range 1, a step change of +8.3 *µ*M (i.e., the entire physiologically relevant range) in the intracellular concentrations of the enzymes and sugar nucleotides of input mode, *µ_7_*, will only result in small changes in the relative percentage of the associated glycans, perhaps as small as 1%. Larger changes to the associated glycan relative percentages would not be possible as this would require step changes to the input concentrations of enzymes and sugar nucleotides that fall outside the allowable range. Conversely, in controlling the first output mode *η_1_* in Range 1, a similar step change to the inputs will result in a much larger change to the relative percentage of the associated glycans, as much as almost 20%. Thus while 7 output modes are controllable, directing *η_7_* to a desired state will require much larger perturbations in *µ_7_* relative to the perturbations in *µ_1_* required to direct *η_1_* to a desired state. The successively smaller singular values *σ_i_* associated with each mode *i*, from 1 to 7 indicates that progressively larger changes are required in *µ_i_* to effect changes in the corresponding *η_i_.*

The magnitudes of the singular values associated with the controllable output modes in Range 2 are comparable to those of Range 1, suggesting that the controllability of the glycan class output modes is similar in these two operating ranges. However, it is important to note that the glycan classes comprising *η_i_* over operating Range 1 are not necessarily the same glycan classes comprising *η_i_* over Range 2 as we now discuss.

#### Relating controllable output modes to glycan classes

As discussed in section “Assessing Controllability from the Process Gain Matrix”, each output mode *η_i_* arising from singular value decomposition of the gain matrix consists of a weighted sum (or linear combination) of the original variables, with the elements of the *i^th^* row of the *W^T^* matrix as the weights (See Eq. 9). How much an original variable contributes to an output mode (a linear combination of glycan classes) is reflected in the coefficient associated with that variable in the linear combination. A dominant contributor to a mode (where one exists) is identified as the variable with the largest coefficient in the weighted sum.

Figure 2 shows the coefficients for variables associated with each glycan class comprising the various controllable output modes in the three operating ranges tested. This figure provides some insight into the characteristics of glycosylation. Most notably, the glycan classes represented in each mode differ greatly from one operating range to another, highlighting the inherent nonlinearity of the glycosylation process. For example, in Range 1, the most controllable mode, *η_1_*, is dominated by the F0 and F1 classes as well as the S0 class (note the scale). The relative coefficient magnitudes within the mode suggest that in Range 1, perturbing the enzyme and sugar nucleotide concentrations in the associated input mode, µ_1_, will affect the F0 and F1 glycan classes much more than the S0 glycan class and the S0 more than any other glycan class. In Range 2, on the other hand, the coefficients associated with most of the contributors to *η_1_* are much smaller, and the group now includes the G0 and G1 classes. The relative equivalence of the coefficient magnitudes for the F0, F1, G0, and G1 classes in output mode, *η_1_*, of Range 2, indicates that in this operating range, each of these glycan classes will be affected approximately equally by changes in the associated input mode. A comparison of the coefficient magnitude scales of the two operating ranges suggests that the process gains associated with *η_1_* in Range 2 are smaller than the process gains associated with *η_1_* in Range 1, which is confirmed by inspection of the process gain matrices (See Figure 1). We also observe that the fourth most controllable mode in Range 2, *η_4_*, while consisting of multiple glycan classes, is entirely dominated by the S1 glycan class; in Range 1, the S1 class barely features in *η_4_* and nowhere else. The fact that the S1 glycan class dominates only one output mode and that output mode is associated with a relatively small singular *σ_4_* = 4.5, suggests that increasing the relative percentage of S1 glycan species may be a difficult control objective to achieve in practice. This is not surprising as the relative percentage of sialylated glycan species of monoclonal antibodies produced in CHO, for instance, is typically low (i.e. <0.5%) [25], [26].

As previously alluded to, the singular values and coefficients associated with each glycan class in a particular output mode provide guidance regarding how best to achieve desired glycan distributions. For example, a process objective to maximize fucosylation in the glycan distribution (i.e., maximize the F1 glycan class), is clearly best achieved via an output mode for which the F1 glycan class is the most dominant contributor. However, multiple output modes are dominated by the F1 glycan class: *η*_1_ and *η*_4_ in operating Range 1, and *η*_1_ in operating Range 2. To select the most appropriate output mode for achieving the process objective, we now recall from section “Identifying controllable modes” that how effectively any mode can be controlled is indicated by the associated singular value. Specifically, output modes associated with larger singular values are more controllable than those associated with smaller singular values. In the particular case in question, we observe that of the three output modes where the F1 class dominates, *η_1_* in operating Range 1 is associated with the largest singular value, *σ_1_* = 39.2. Therefore, to maximize the F1 glycan class, one should restrict the enzyme and sugar nucleotide donor concentrations to the Range 1 values and use input mode *µ_1_* to control output mode *η_1_*.

On the other hand, if the process objective is to maximize the relative percentage of glycan species in the glycan distribution with both branches galactosylated (i.e., maximize the G2 glycan class), the best option is to operate the process in Range 2 and use input mode *µ*_5_ to control output mode *η*_5_. This is because while output mode *η*_5_ in Range 2 is co-dominated by the S2 and G2 glycan classes with coefficients 0.6 and -0.6 respectively, it is the only controllable output mode where the G2 class is at least partially dominant. (Output mode *η*_7_ in Range 2, barely controllable with *σ_7_* = 1.0, consists of many other glycan classes, such as S0, S2 and S3, whose contributions to the mode are almost as significant as that of G2.) The relatively small singular value associated with *η*_5_ in Range 2, *σ*_5_ = 2.52, indicates that even with this choice, maximizing the G2 glycan class may be a relatively difficult process objective to achieve in practice.

These concepts are also useful when considering multiple simultaneous process objectives, such as, say maximizing both the F1 and G2 glycan classes. The controllable output modes in operating Range 1 involve only the fucosylated and sialylated glycan classes, so that only these two glycan classes can be directed to a desired state if the process is operated in this range. On the other hand, the controllable modes in operating Range 2 provide some degree of control over all the glycan classes listed in Table 1 except the S4 and G4 classes. The simultaneous objective of maximizing the F1 and G2 glycan classes may therefore be met via the dual strategy of (i) directing the F1 glycan class to a desired state by using input mode *µ*_1_ to control output mode *η*_1_ in operating Range 2 (rather than Range 1); and (ii) using input mode µ_5_ to control output mode *η*_5_ in operating Range 2, since, as discussed above, the G2 glycan class is theoretically controllable only via this option. Finally, observe that the singular value for output mode *η_1_* in operating Range 2, *σ_1_* = 25.9, is technically smaller than the corresponding singular value, *σ_1_* = 39.2 in Range 1; practically, however, both singular values are of comparable magnitude and thus will afford similar degrees of control for the F1 glycan class. Therefore by operating in Range 2, both F1 and G2 can be directed jointly to respective desired states, whereas in operating Range 1, only F1 would be controllable.

#### Relating input modes to glycosylation enzyme and sugar nucleotide donor concentrations

The process controllability analysis presented here involves more than just the determination of controllable output modes (linear combinations of glycan classes); it also identifies the appropriate corresponding input mode that can be used to affect the most change in an output mode such that the output mode can be directed effectively and efficiently to a desired state. Just as each output mode *η_i_* is a linear combination of the glycan classes, each input mode *µ_i_* is also a linear combination of the original inputs variables (i.e., glycosylation enzymes and sugar nucleotide donor concentrations). And as with the output modes, elements of the *i^th^* row of the *V^T^* matrix are the coefficients of the weighted sum of original inputs that make up the *µ_i_* mode (see eq. 10).

Figure 3 shows the coefficients associated with the 15 glycosylation enzyme and sugar nucleotide donors that make up the controllable input modes, *µ_i_*, for each operating range investigated. Each *µ_i_* is a unique combination of glycosylation enzyme and sugar nucleotide donors that can be used to influence the glycan classes of each corresponding output mode *η_i_*. In all operating ranges investigated, the majority of coefficients associated with sugar nucleotide donors are zero. A comparison of the coefficients associated with sugar nucleotide donors against those associated with the glycosylation enzymes (the latter of which are larger on average, and across the board), suggests that glycosylation enzyme concentrations may have a *greater impact* on the resulting glycan distribution than sugar nucleotide donor concentrations. This result is supported by the data of Hills *et al* (2001), which showed that increasing intracellular UDP-Gal 5 fold did not increase the degree of galactosylation significantly, and increasing CMP-Sialic Acid by 44-fold did not increase sialylation. We hypothesize that mechanistically, the glycosylation enzymes are rate limiting with respect to the sugar nucleotide donor concentrations within the Golgi apparatus.

### Controlling Specific Glycoforms

Thus far, results of the controllability analysis suggest that multiple controllable output modes exist for glycan classes when the operating conditions are restricted to the Range 1 and 2 values. However, rather than influencing entire classes of glycans, a more desirable, albeit challenging, control objective would be to direct the relative percentage of *specific* individual glycoforms from some initial state to some desired final state such that the glycan distribution of a therapeutic protein batch will consist of only a few, or perhaps even a single, specific glycoform. The ability to achieve such a control objective would substantially reduce glycan heterogeneity, which, in turn, may help to improve the consistency of the therapeutic protein's pharmacokinetic behavior.

**Figure 4:**
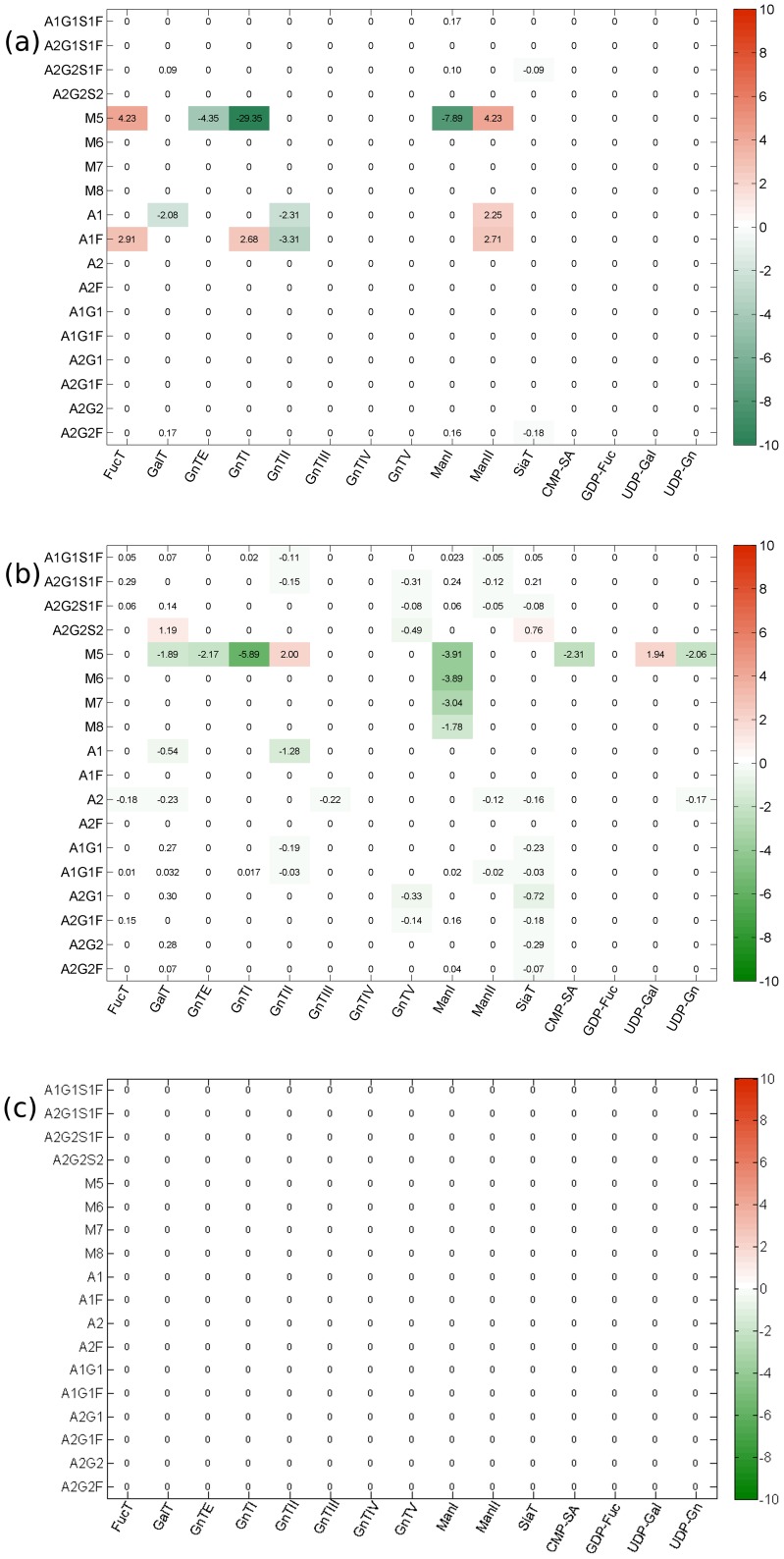
Heat maps representing the significant elements of the process gain matrices for specific glycoforms typically found in biologics in operating (a) Range 1, (b) Range 2, and (c) Range 3. Visual inspection suggests that significant process gains are associated with 8 of the 18 glycoforms when the process is operated in Range 1 and 11 of the 18 glycoforms when operated in Range 2, indicating that the relative percentage of glycoforms can be changed in these operating ranges. As with the glycan classes, there are no significant process gains for any glycoforms in operating Range 3, suggesting that the relative percentage of glycoforms cannot be affected or controlled at all when the process is operated in Range 3.

The extent to which individual glycoforms can be directed from some initial state to some desired final state, with the available enzymes and sugar nucleotide donors, can be determined from appropriate glycoform controllability analysis. For the specific example in question, applying the controllability analysis method to the specific glycoforms listed in Table 2 produced the following results. First, Figure 4 shows a heat map of the relevant process gains. Visual inspection indicates that significant process gains are associated with 8 of the 18 glycoforms when the process is operated in Range 1, and 11 of the 18 glycoforms when operated in Range 2. There are no significant process gains for any glycan class in operating Range 3, suggesting that, as with the glycan classes, one cannot achieve any arbitrarily desired relative percentage of specific glycoforms when the process is operated in Range 3. A general comparison with Figure 1 (for glycan classes) indicates that even the specific glycoforms that are controllable will be more difficult to control than glycan classes because the former on average have comparatively smaller process gains.

**Figure 5:**
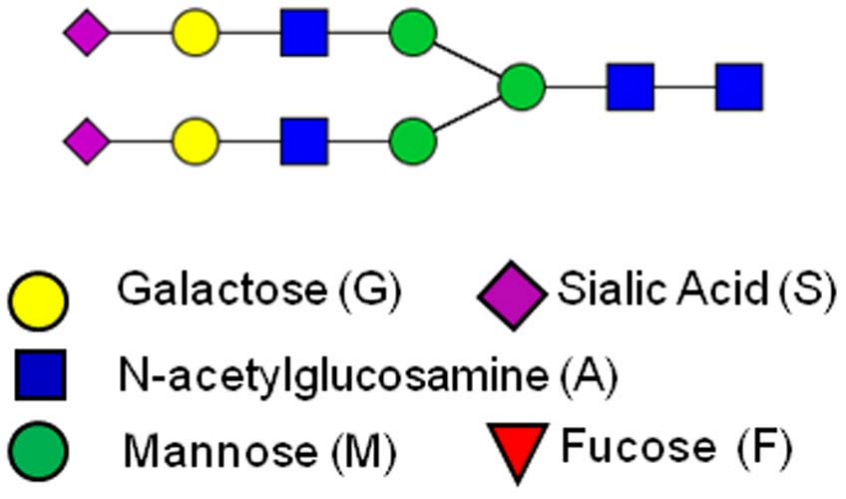
Example glycoform structure – A2G2S2 glycoform.

We illustrate the controllability of specific glycoforms using as a representative case the A2G2S2 glycoform which, as shown in Figure 5, has two terminal sialic acid molecules. It has been shown [7] that increasing the relative amount of terminal sialic acid increases serum half-life. Thus, one way to prolong the pharmacokinetic effects of a therapeutic treatment *in vivo* may be to produce the therapeutic protein in question with predominantly the A2G2S2 glycoform attached.

Visual inspection of the significant process gains in Figure 4 over the 3 ranges of operating conditions investigated in this controllability analysis indicates that controllable modes exist for specific glycoforms when the process operation is restricted to Ranges 1 and 2 but not 3. Specifically for the A2G2S2 glycoform, however, only Range 2 contains significant, non-zero process gains, suggesting that controllable modes exist for the A2G2S2 glycoform only in operating Range 2.

The singular values shown in Table 4, obtained from singular value decomposition of the process gain matrices, indicate that 3 of the 18 output modes are controllable in Range 1, and 5 of the 18 output modes are controllable in Range 2. As was the case with the glycan classes, none of the output modes in Range 3 is controllable.

**Table 4:** Singular values, *σ_i_*, obtained from singular value decomposition of the glycoform process gain matrix for three operating ranges.

Singular Values	Range 1	Range 2	Range 3
**σ_1_**	31.3	9.1	0
**σ_2_**	6.2	4.4	0
**σ_3_**	2.5	1.6	0
**σ_4_**	0.3	1.2	0
**σ_5_**	0.1	0.9	0
**σ_6_**	4.5E-4	0.5	0
**σ_7_**	2.7E-16	0.4	0
**σ_8_**	9.7E-18	0.2	0
**σ_9_**	2.4E-21	0.1	0
**σ_10_**	1.2E-33	1.4E-1	0
**σ_11_**	6.9E-37	2.3E-16	0
**σ_12_**	0	1.3E-16	0
**σ_13_**	0	6.1E-17	0
**σ_14_**	0	2.1E-17	0
**σ_15_**	0	4.6E-33	0
**σ_16_**	0	0	0
**σ_17_**	0	0	0
**σ_18_**	0	0	0

Output modes associated with singular values, *σ_i_*>σ_*_ = 1, are considered controllable.

**Figure 6:**
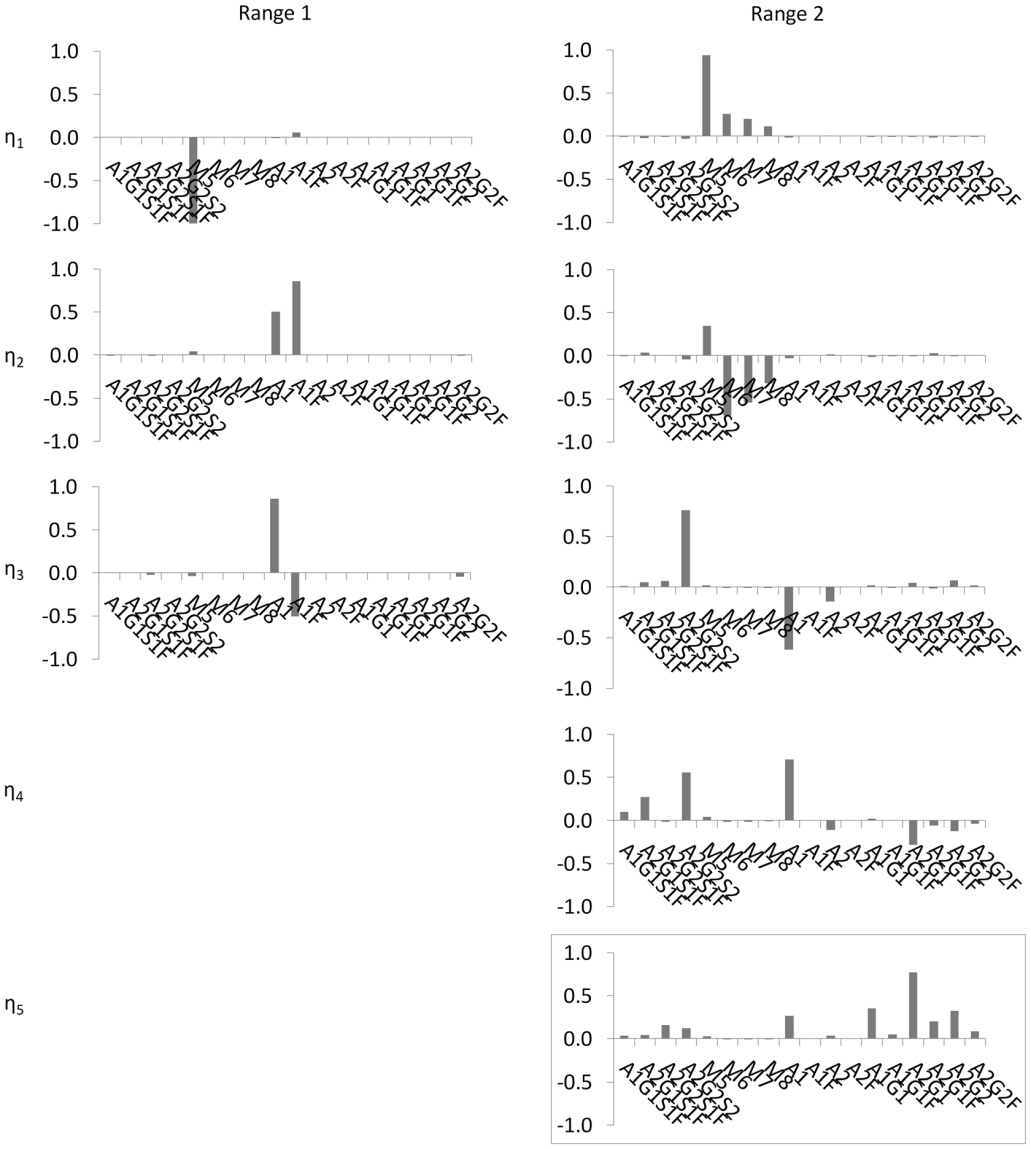
Graphical representation of the coefficients associated with each glycoform in the controllable output modes, *η_i_.* Modes that were not controllable (i.e. associated with singular values, ***σ_i_*** **<**σ_*_ = 1) are not shown. Columns show the glycoforms of each controllable output mode in each operating range (See Table 1). No output modes are shown for operating Range 3 since no controllable modes were found in this range. Coefficients were obtained using eq. 9 following singular value decomposition of the glycoform process gain matrix as described in section “Assessing Controllability from the Process Gain Matrix”. How much the glycoform contributes to an output mode is reflected in the coefficient associated with that variable in the linear combination. A dominant contributor to a mode (where one exists) is identified by the variable with the largest coefficient in the weighted sum. Any glycoform associated with a non-zero coefficient can be affected by perturbations in the associated input mode; however the dominant glycoforms will be affected the most.

**Figure 7:**
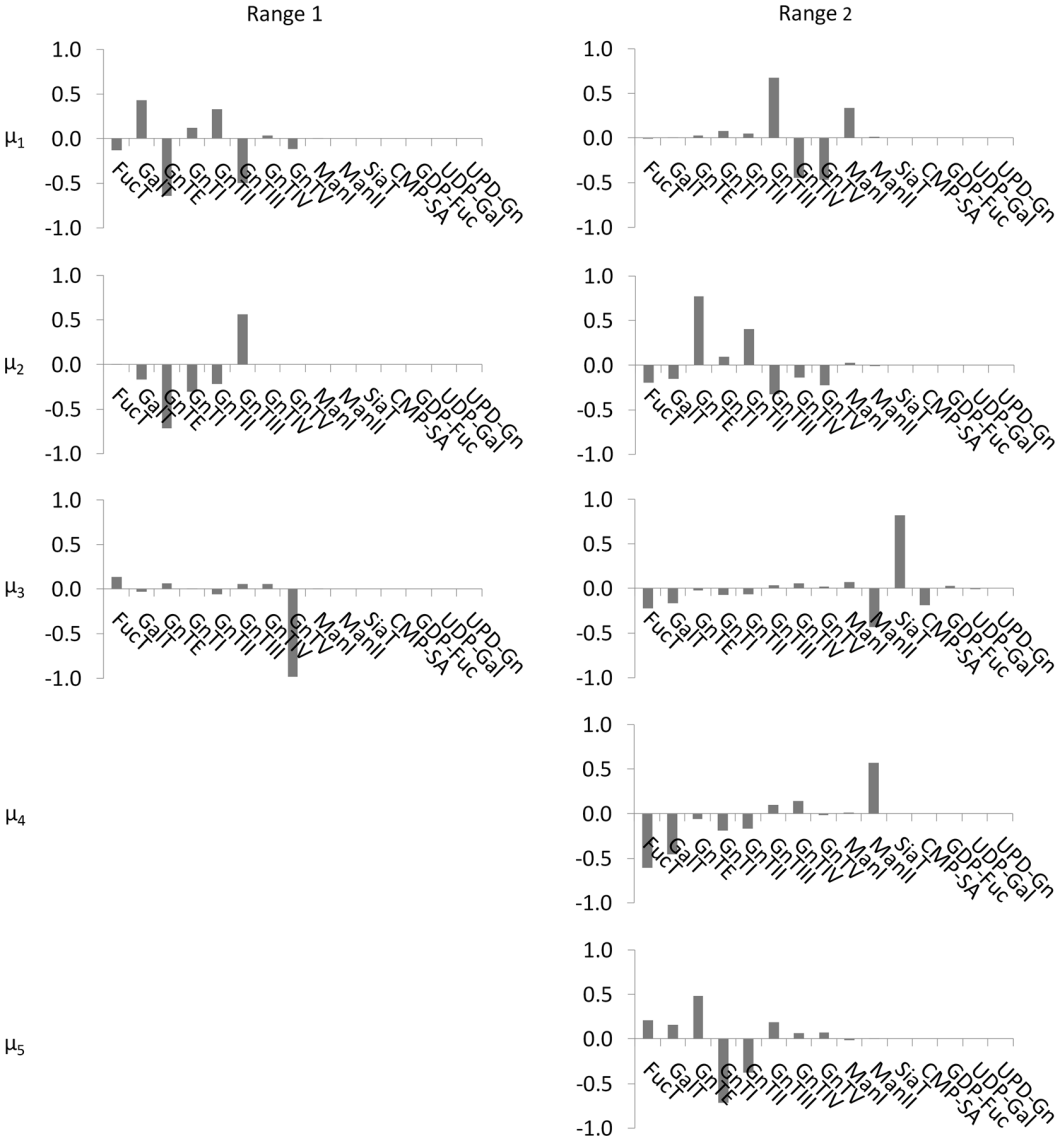
Graphical representation of the coefficients associated with each enzyme and sugar nucleotide donor in input modes, µ*_i_*, associated with the controllable output modes for glycoforms, *η_i,_* shown in Figure 6. Each column shows the glycosylation enzymes and sugar nucleotides of each input modes in each operating range (see Table 1). No input modes are shown for operating Range 3 since no controllable modes were found in this range. Coefficients were obtained using eq. 10 following singular value decomposition of the process gain matrix corresponding to the glycoform distribution as described in section “Assessing Controllability from the Process Gain Matrix”. How much the enzyme or sugar nucleotide contributes to an input mode is reflected in the coefficient associated with that variable in the linear combination. A dominant contributor to a mode (where one exists) is identified by the variable with the largest coefficient in the weighted sum. The enzyme(s) and/or sugar nucleotide(s) that are dominant contributors of the input mode affect the glycoforms of the associated output mode the most.

Figure 6 shows a graphical representation of coefficients of glycoforms comprising each controllable output mode, *η_i,_* while Figure 7 represents the coefficients of enzyme and sugar nucleotide donors comprising each input mode, *µ_i_* graphically. The coefficients of *η_i_* suggest that the A2G2S2 glycoform dominates two output modes *η_3_* and *η_4_* over Range 2, but, as already inferred from the gain matrix, this glycoform is not represented in any output mode in Range 1 or 3. Of the two output modes in Range 2 where the A2G2S2 is the dominant contributor, *η_3_* is associated with the largest singular value. Consequently, to control the A2G2S2 glycoform the operating conditions should be restricted to the Range 2 values and input mode µ_3_ (See Figure 7) should be used to control output mode *η_3_* (See Figure 6).

In addition to controlling the A2G2S2 glycoform, one may wish to direct other glycoforms simultaneously to respective desired states in order to induce some other pharmacokinetic effects *in vivo*. In such a case, the controllability results provide useful insight into which glycoforms are controllable in a particular operating range. Specifically, observe that when the enzymes and sugar nucleotide donors are restricted to the Range 2 values, not only is the A2G2S2 glycoform controllable with the available controllable output modes, but each sialylated, high mannose glycoform, as well as G1 and G2 glycoform, are also controllable (See Figure 7). Conversely, the output modes in Range 2 provide limited or no control over the A1F and A2F species. Thus if the desired (or undesired) pharmacokinetic behavior results from the presence or absence of A1F or A2F species, it is not beneficial to restrict the glycosylation enzymes and sugar nucleotide donors to Range 2. On the other hand, operating Range 2 is ideal for producing proteins whose therapeutic characteristics depend on the presence of the A2G2S2 glycoform jointly with any or all of the sialylated, high mannose glycoform or G1 and G2 glycoforms.

### Implications of Controllability Analysis

By applying the controllability analysis method described in this work to the glycosylation process, we have identified the conditions under which glycosylation is controllable (i.e., we have identified the operating ranges for which controllable input and output modes exist), and the extent of controllability, (i.e., the glycosylation control objectives that are achievable in practice). We have also shown that due to the non-linearity of the glycosylation process, process controllability characteristics change over different operating ranges (i.e., different ranges of intra-Golgi glycosylation enzyme and sugar nucleotide donor concentrations)—with the practical implication that some operating ranges are more conducive than others for achieving particular glycosylation control objectives. As such, these results have provided insight into which specific operating regimes are most conducive to meeting certain product quality objectives (e.g., glycan distributions), and how best to meet these objectives by identifying the input modes (relative intracellular concentrations of enzymes and sugar nucleotides) that can be used to bring about the most efficient change to the desire output mode. It is imperative to note, however, that the specific results from this study depend on the fidelity with which the glycosylation process is faithfully represented by the model. The glycosylation process may vary by cell line and production process. Therefore the mathematical representation of the glycosylation process should be validated by experimental data prior to employing controllability analysis for process design decisions.

For process control purposes, the intent of controllability analysis is to identify operating regions where the extent of controllability is relatively high so that desired changes in the output variables can be effected relatively easily. However, identifying operating conditions of low controllability is also useful, but for different reasons. Consider the case where the desired glycan distribution can be achieved in a few different operating regions, one of which is a “low controllability” region. Because with low controllability the process output variables cannot be changed to any significant extent by changes in the input variables, observe that the process will essentially be immune (hence robust) to fluctuations in the values of the input variables in such “low controllability” regions. Thus, for robust design purposes, the objective is to identify a region (in which to operate the process) where the product quality objectives can be met and which is simultaneously of “low controllability”. Under such conditions, the process can operate robustly without much active control, and quality will be achieved consistently by design.

The controllability analysis method we have described also has clear implications for cellular engineering applications. The controllability analysis identifies what combination of glycosylation enzymes and sugar nucleotides will produce a particular glycan distribution. Therefore, the results can be used to guide genetic engineering of cell lines. Prior to the analysis presented here, the indirect and interactive effects of glycosylation enzymes on final glycosylation distribution were largely unknown. While there have been attempts to engineer cell lines that yield a desired glycan distribution, past attempts have generally focused on the over expression or deletion of only one or a few glycosylation enzymes without a concrete *a priori* knowledge of the effects on the resulting glycan distribution [27]–[30]. Such fortuitous attempts are effective for identifying the function of enzymes; however, a more directed and systematic approach is required if one wishes to design a cell line that has the capability to produce a specific desired glycan distribution. The controllability analysis we have presented here is a tool that can be used to provide a more systematic approach to cellular engineering.

Media formulation can also be guided by controllability analysis. Media formulation and media supplements also have been shown to affect the glycan distribution by altering the glycosylation enzyme expression and intracellular sugar nucleotide donor concentration [25], [31]–[33]. Again, with these media supplement studies, glycan distributions are altered in an ad-hoc fashion, with no concrete *a priori* knowledge. These studies have been useful for identifying the specific media components that affect the relative percentage of certain glycans and, in some cases, the intracellular mechanisms that result in such effects. However, to direct the glycan distribution to a desired state via a media formulation approach, it would be beneficial to identify which combination of supplements will result in the desired glycan distribution prior to performing any experimental media studies. The controllability analysis we discuss identifies which combination of enzymes *should* be manipulated and in what manner in order to produce a desired glycan distribution. With the knowledge of how media supplements affect enzyme and sugar nucleotide concentrations, controllability analysis can be used as a tool to predict rationally the media formulation required to achieve a particular glycosylation control objective (or any quality attribute for that matter) and, as such, guide a more effective media development process.

## Conclusions

In this work, we have developed a methodology for assessing the controllability of glycosylation, a prototypical example of an extremely high-dimensional and very non-linear system. The method involves first obtaining the process gain matrix via simulation, carried out using a DOE strategy, followed by singular value decomposition of the process gain matrix from which the controllability may be assessed. The practical utility of the method was demonstrated with an illustrative example in which controllability was assessed for various classes of glycans as well as specific glycoforms that are typically found in recombinant biologics produced with CHO cells.

While the specific results from this study depend on the fidelity with which the glycosylation process is faithfully represented by the model, (which may vary by cell line and production process), this work has provided, for the first time, a systematic method for determining, in a quantitative manner, the intrinsic controllability of the glycosylation process. And as noted above, these results also have implications for rational cellular engineering and media formulation design. Once the best possible system (rationally engineered cell-line, combined with the operating conditions conducive to achieving the desired objective) is obtained through process design, and the best achievable control is determined through controllability analysis, the next step is to develop a control system that can achieve the glycosylation control objectives consistently and reproducibly.
